# Enhancement of the Transmission Performance of Piezoelectric Micromachined Ultrasound Transducers by Vibration Mode Optimization

**DOI:** 10.3390/mi13040596

**Published:** 2022-04-10

**Authors:** Penglu Li, Zheng Fan, Xiaoya Duan, Danfeng Cui, Junbin Zang, Zengxing Zhang, Chenyang Xue

**Affiliations:** Key Laboratory of Instrumentation Science & Dynamic Measurement, Ministry of Education, North University of China, Taiyuan 030051, China; lipenglu98@163.com (P.L.); s1906119@st.nuc.edu.cn (Z.F.); duanxiaoya11111@163.com (X.D.); zangjunbin@nuc.edu.cn (J.Z.); zhangzengxing@nuc.edu.cn (Z.Z.)

**Keywords:** PMUT, FEM, vibration diaphragm, high frequency

## Abstract

Ultrasound is widely used in industry and the agricultural, biomedical, military, and other fields. As key components in ultrasonic applications, the characteristic parameters of ultrasonic transducers fundamentally determine the performance of ultrasonic systems. High-frequency ultrasonic transducers are small in size and require high precision, which puts forward higher requirements for sensor design, material selection, and processing methods. In this paper, a three-dimensional model of a high-frequency piezoelectric micromachined ultrasonic transducer (PMUT) is established based on the finite element method (FEM). This 3D model consists of a substrate, a silicon device layer, and a molybdenum-aluminum nitride-molybdenum (Mo-AlN-Mo) sandwich piezoelectric layer. The effect of the shape of the transducer’s vibrating membrane on the transmission performance was studied. Through a discussion of the parametric scanning of the key dimensions of the diaphragms of the three structures, it was concluded that the fundamental resonance frequency of the hexagonal diaphragm was higher than that of the circle and the square under the same size. Compared with the circular diaphragm, the sensitivity of the square diaphragm increased by 8.5%, and the sensitivity of the hexagonal diaphragm increased by 10.7%. The maximum emission sound-pressure level of the hexagonal diaphragm was 6.6 times higher than that of the circular diaphragm. The finite element results show that the hexagonal diaphragm design has great advantages for improving the transmission performance of the high-frequency PMUT.

## 1. Introduction

Ultrasound refers to a sound-wave signal with a frequency higher than 20 kHz [1]. Ultrasound has the advantages of strong penetrating ability, good directionality, and easy concentration of sound energy; it is therefore used in many fields, such as medicine, industry, and agriculture [2]. Ultrasonic transducers are the key components of ultrasonic applications, which can achieve the mutual conversion of mechanical energy and electrical energy [3]. Traditional bulk ultrasonic transducers have some disadvantages [4,5], being incompatible with ICs and difficult to form arrays. In recent years, with the development of MEMS technology, MEMS-based ultrasonic transducers, namely micromachined ultrasonic transducers (MUTs), have emerged [6,7]. Compared with traditional bulk ultrasonic transducers, MUTs have the advantages of small size, light weight, low power consumption, high reliability, easy frequency control, high sensitivity, and easy integration with circuits [8,9]. MUTs can be divided into two types: capacitive micromachined ultrasonic transducers (CMUTs) [10,11,12,13], and piezoelectric micromachined ultrasonic transducers (PMUTs) [14,15,16]. Compared with CMUTs, PMUTs have the advantages of being compatible with CMOS [17,18], low driving voltage, and easy arrayment. PMUTs are increasingly used in ultrasonic ranging, non-destructive testing, parking radar, medical imaging [19], and fingerprint recognition [20], among other applications.

In 2011, Richard J. Przybyla [21] designed an AlN PMUT based on pulse-echo time-of-flight air ranging, in which a single sensor can transmit and receive sound waves. The ranging range can reach 30-450 mm. Yipeng Lu [22,23] (2015) designed a high-fill, high-frequency PMUT that can be used for intravascular ultrasound imaging and other medical imaging applications. Xiaoyue Jiang [24,25] (2017) designed a 591 × 438-DPI ultrasonic fingerprint sensor. By reducing the element spacing, PMUT with a high fill factor array was produced, and good acoustic performance was obtained. Changhe Sun [26] (2019) designed a single-element multi-frequency ultrasound transducer that showed significant potential in ultrasound detection, diagnostic and imaging applications, and wireless acoustic energy transfer. Wenjuan Liu [27] (2019) established a 3D model of a high-frequency piezoelectric micromachined ultrasonic transducer based on the finite element method, which is helpful for the design analysis of compact ultrasonic systems. Priya S. Balasubramanian [28] (2020) designed a chip-scale device consisting of piezoelectric aluminum nitride ultrasonic transducers for the GHz-level ultrasonic stimulation of human nerve cells. Alexandre Robichaud [29] (2021) designed a packaged ultrasonic ranging system consisting of a 4–8-PMUT matrix and an interface integrated circuit (IC). The research on PMUTs has become increasingly extensive.

An optimal design can improve the transmission performance, sensitivity, and emission sound-pressure level of a transducer. Many research methods have been used to improve the performance of transducers, including electrode design, etching trenches, and array-structure optimization. Sina Akhbari (2015) [30] designed a two-electrode bimorph transducer. The PMUT achieves higher output sound pressure through differential driving. In 2017, Guo-Lun Luo [31] fabricated high-fill-factor AlN PMUT arrays on transparent substrates. Resonant frequencies from 3 MHz to 18 MHz were generated in the air, achieving a high fill rate of 62%. In 2021, Xuying Chen [32] produced a dual-frequency-excitation PMUT by designing a dual-electrode trench structure, which can achieve high-sensitivity PMUT emission and reception at 103 KHz and 210 KHz. However, there are few studies on the design and comparison of different diaphragm shapes to change the vibration mode of the transducers, thereby improving the performance of PMUTs.

In this paper, the design of an air-backed circular PMUT is presented. According to this structure, square and hexagonal diaphragms were designed for the PMUT. The key dimensions of the three structures were optimized and compared, and the optimal structure and parameter design scheme were determined. The PMUT vibration mode was optimized for higher transmission performance, sensitivity, and emission sound-pressure level.

## 2. Materials and Methods

### 2.1. The Basic Structure and Working Principle of PMUT

A PMUT is a MEMS device. The positive and negative piezoelectric effects of piezoelectric materials make the piezoelectric film vibrate, which allows it to transmit or receive ultrasonic signals. Piezoelectric materials perform acoustic-electric conversion and act as vibration components in the transducer. When it is used as a transmitter, an alternating voltage is applied to the upper and lower surfaces of the piezoelectric layer of the transducer. The piezoelectric layer produces a transverse inverse piezoelectric effect and extends or contracts in the radial direction. However, the vibrating layer (Si) connected to the piezoelectric layer has no piezoelectric effect, resulting in the elongation or contraction of the thin plate. When the piezoelectric layer contracts radially, the entire plate bends downward. The thin plate moves up and down periodically due to the application of alternating current. At this point, it can radiate sound wave signals to achieve the conversion of electrical energy into mechanical energy, and it is an actuator. When it is used as a receiver, the ultrasonic signal acts on the piezoelectric film, and the film produces lateral strain under the action of the piezoelectric effect. The membrane vibrates up and down to produce the conversion of electrical energy to mechanical energy. At this point, it is a sensor.

Currently, the piezoelectric materials commonly used in the MEMS field include lead zirconate titanate (PZT) [33], zinc oxide (ZnO) [34], and AIN [35]. Among these, PZT offers the best piezoelectric performance, higher piezoelectric coefficient, the best low-frequency characteristics, and higher sensitivity. However, the piezoelectric material PZT contains lead, and its processed devices are relatively large in size, low in integration capability, and incompatible with the complementary metal-oxide-semiconductor (CMOS) process. The manufacture of ZnO has the drawback of contaminating the CMOS process. AlN piezoelectric film is an environmentally friendly lead-free piezoelectric material that can be used in implantable medical devices. In addition, AlN has a lower dielectric constant and ten times higher receiving sensitivity than PZT piezoelectric ceramic films. Its compatibility with the CMOS process, ability to maintain piezoelectric properties at high temperatures (<900 °C), suitability for mass manufacturing, and good acoustic matching make AlN an excellent material for designing structures.

**Figure 1:**
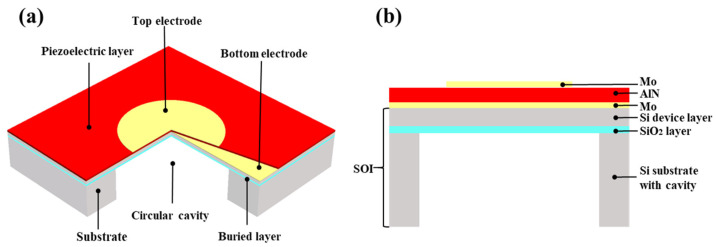
(**a**) Schematic diagram of the three-dimensional structure of PMUT; (**b**) cross-sectional schematic diagram of the three-dimensional structure of PMUT.

Figure 1 shows the three-dimensional model of the sensor, which can be roughly divided into two parts: the upper piezoelectric vibrator and the lower substrate. The upper piezoelectric vibrator is the key vibration film unit for the acoustic-electric conversion, which is composed of a SOI buried oxygen layer (SiO2), a SOI device layer (Si), and a sandwich structure composed of a Mo-AlN-Mo sensitive layer. The piezoelectric film is sandwiched between the upper and lower electrodes, and the electrodes are used to collect the charges generated by the piezoelectric film. If the lattice mismatch between the electrode and the piezoelectric film is large, the lattice of the film is distorted, resulting in internal stress and easy cracking. Therefore, it is necessary to select suitable electrode materials to grow AlN piezoelectric thin films. At present, the most commonly used electrode materials are Pt, Au, Al, Ti, and Mo, among which Ti and Mo have the closest lattice constants to AlN, but Ti electrodes have the drawbacks of difficulty in patterning and high resistivity. Since molybdenum has low acoustic attenuation, good electrical conductivity, good adhesion to AlN, and easy patterning, it is more suitable for the fabrication of acoustic sensors, and the adhesion between AlN and Mo is good. In addition, the acoustic impedance of Mo is higher than that of AlN, and Mo has lower resistance and lower material loss within the same device structure. As the bottom electrode, the device has the highest quality factor. The substrate under the bottom electrode mainly plays a supporting and fixing role, which can increase the reliability of the design and increase the resonance of the device. The SOI backside is etched to the buried oxide layer using deep reactive ions to release the vibrating film. The corresponding part of the suspended area of the back cavity is the diaphragm, and the cavity adopts the form of air coupling. The key structural dimensions of the device are shown in Table 1.

### 2.2. Membrane Vibration Equation

A tight planar film is in the equilibrium position when the film lies in the xy plane. When the membrane is disturbed by an external force perpendicular to the xy plane, the membrane deforms, either in a concave or a bulge shape. Under the action of the tension (T), transverse vibration in the vertical direction is generated. Let η be the vertical displacement of a point on the membrane away from the equilibrium position, that is, the displacement of the membrane. Take a one-sided element dxdy on the membrane. When the surface element is deformed, the membrane at its edge is subject to the tension of the adjacent element segments. The vertical force acting on the entire panel can be expressed as [36]:

(1) $$F_{T}=T(\frac{\partial^{2}\eta }{\partial x^{2}}+\frac{\partial^{2}\eta }{\partial y^{2}})dxdy$$

According to Newton’s second law, the equation of motion of the film can be obtained as [37]:

(2) $$\nabla^{2}\eta =\sigma dxdy(\frac{\partial^{2}\eta }{\partial t^{2}})=\frac{1}{c^{2}}\frac{\partial^{2}\eta }{\partial t^{2}}$$

In the formula, σ is the mass of the film per unit area, σdxdy is the areal density, $c=\sqrt{\frac{T}{\sigma }}$, and $\nabla^{2}=\frac{\partial^{2}}{\partial x^{2}}+\frac{\partial^{2}}{\partial y^{2}}$ is the Laplace operator of the two-dimensional rectangular coordinate system.

For the plane circular film with a fixed edge, polar coordinate form is adopted: x=acosθ, y=asinθ. Here, a is the polar diameter of the film and θ is the angle between the polar diameter and the polar axis. Since the circular diaphragm vibrates in a symmetrical form, the vibration displacement is only a function of the radial distance (a). Substituting this into Equation (1), the vibration equation can be obtained as:

(3) $$\frac{\partial^{2}\eta }{\partial a^{2}}+\frac{1}{a}\frac{\partial \eta }{\partial a}=\frac{1}{c^{2}}\frac{\partial^{2}\eta }{\partial t^{2}}$$

Using the separation variable method, the displacement of the membrane during vibration can be obtained as [38,39,40]:

(4) $$\eta (t,a)=AJ_{0}(ka)e^{jwt}$$

where $T(t)=e^{jwt}$, $k=\frac{w}{c}$, A is a constant, and $J_{0}$ is a zero-order cylindrical Bessel function.

### 2.3. Resonant Frequency

The propagation of ultrasonic waves in the medium has attenuation, and the design of the operating frequency of the transducer is an important factor that affects the attenuation of ultrasonic energy by the acoustic medium. Many important properties of the transducer, including its receiving sensitivity, directivity, transmitting sound power, and sound field characteristics, are directly affected by its operating frequency. Thus, when determining the operating frequency of the device, it is necessary to comprehensively consider and take into account the influencing factors of various aspects. Generally speaking, the best working state, electromechanical conversion efficiency, and emission sound-pressure level can be obtained when the transmitting transducer works at its resonant fundamental frequency. Furthermore, under this condition, the best response and receiving sensitivity can be obtained by a receiving transducer. The first-order resonant frequency of a circular PMUT can be expressed as [41]:

(5) $$f_{0}=\frac{40.8\times t}{2\pi \times a^{2}}\sqrt{\frac{E}{12\times \rho \times (1-v^{2})}}$$

The flexural stiffness of the N-layer membrane is defined as [37]:

(6) $$D=\int \frac{E_{n}}{\left(1-\upsilon_{n}^{2}\right)}z^{2}dz=\frac{1}{3}\sum_{n=1}^{N}\frac{E_{n}}{\left(1-\upsilon_{n}^{2}\right)}\left({\bar{h}}_{n}^{3}-{\bar{h}}_{n-1}^{3}\right)$$

where $E_{n}$, $\rho_{n}$, and $v_{n}$ are the Young’s modulus, density, and Poisson’s ratio of the nth layer, respectively, ${\bar{h}}_{n}^{}$ is the distance from the top of the nth layer to the neutral plane of $Z_{N}$ the multilayer film, and z is an integral variable. The neutral plane position $Z_{N}$ of the N-layer film is defined as [42,43]:

(7) $$Z_{N}=\frac{\sum_{n=1}^{N}t_{n}z_{n}E_{n}}{\sum_{n=1}^{N}t_{n}E_{n}}$$

where $t_{n}$ is the thickness of the nth layer and $z_{n}$ is the position of the nth layer relative to the bottom layer.

The resonant fundamental frequency of the circular membrane vibration transducer is inversely proportional to the radius, which can be obtained through Equation (5).

### 2.4. Sensitivity

A sensor’s sensitivity is an important indicator of its performance, reflecting the output response per unit of input sound pressure. The received voltage sensitivity of a transducer is the ratio of the sensor’s output to another specific input. In other words, it is the ratio of the open-circuit voltage U at the output end to the free-field sound pressure P at the acoustic center of the receiving surface before the transducer works.

(8) $$M=\frac{U}{P}\;(V/\mathrm{Pa})$$

Expressed in decibels, the received voltage sensitivity level of the transducer can be expressed as:

(9) $$S=20\mathrm{lg}\frac{M}{M_{r}}(M_{r}=1\;V/\mu \mathrm{Pa})$$

### 2.5. Transmit Voltage Response Level

The emission voltage response is the ratio of the product of the transient sound pressure and the reference distance to the input voltage at a certain frequency or in a specific direction. It can be expressed as:

(10) $$S_{v}=\frac{p_{0}\cdot d_{0}}{v}$$

The unit is Pa⋅m/V. Here, $d_{0}$ is the distance from the reference point of the acoustic center of the transmitting transducer, and the reference distance is 1 m. The emission voltage response is expressed in decibels as the emission voltage response level, which can be expressed as:

(11) $$S_{l}=20\mathrm{lg}\frac{S_{v}}{S_{r}}(S_{r}=1\;\mathrm{Pa}\cdot m/V)$$

## 3. Finite Element Analysis

### 3.1. Proposed Structure

**Figure 2:**
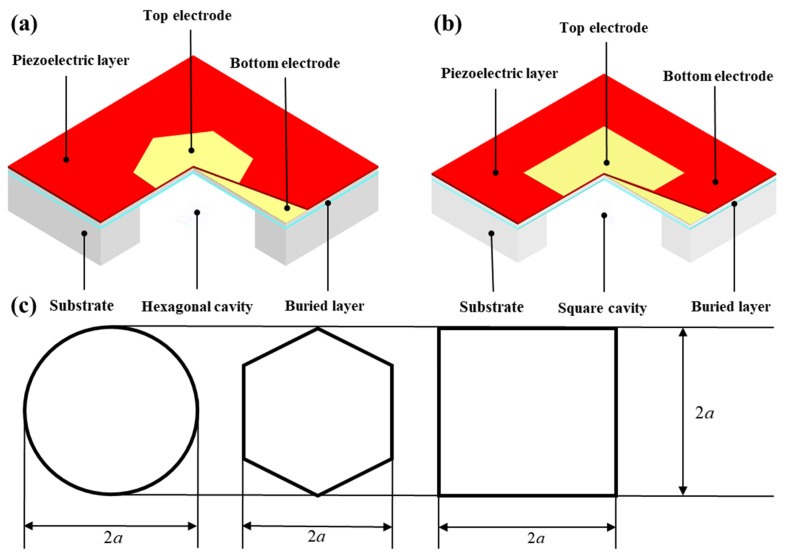
Structure of the PMUT. (**a**) Hexagonal-diaphragm PMUT; (**b**) square-diaphragm PMUT; (**c**) dimensional relationship of three PMUT structures.

The vibration mode of the transducer plays an important role in improving the performance of the device. Different vibration modes of the transducer at the resonant frequency can be achieved by changing the shape of its diaphragm. Based on the traditional circular plate, hexagonal and square thin-film sensors were designed, as shown in Figure 2a,b. The diameter of the circle, the diameter of the circumcircle of the regular hexagon, and the side length of the square are equal; denoted by 2a. The critical dimension relationships of the three structures are shown in Figure 2c. Hexagonal and square shapes are both simple shapes with edge distribution rules. When transducers are designed as an array, it is easy to achieve a high filling factor.

### 3.2. Modal Analysis

The finite element model of the piezoelectric film was established using COMSOL multiphysics software to analyze the effect of the piezoelectric thin-film transmission sensitivity. The structural modal is the natural vibration characteristic of the structural system, which is only related to the inherent properties of the structure, including mass, shape, material, etc., and has nothing to do with the medium. Therefore, the simulations in this chapter were carried out in COMSOL’s default ideal medium, which is a vacuum environment. The material parameters used in the simulation are shown in Table 2.

The performance of the transducer mainly depends on the vibrating membrane, which is independent of the substrate. To reduce the amount of calculation and save computing resources, only the vibration membrane was constructed in the geometry during modeling. Since the symmetric boundary conditions did not apply to the hexagonal films, a 3D model was used for simulation analysis. By fixing the boundary conditions, the support of the substrate for the vibrating membrane was simulated. After simplifying the model, the silicon of the device layer was a key part of the diaphragm and did not need to be etched. The effect of the isotropic and anisotropic silicon on the results was not significant. Therefore, the default isotropy was used. The thickness of the upper electrode was less than that of the whole vibrating film, which had little influence on the vibration performance of the device. Therefore, the upper electrode was simplified to a complete vibration model for analysis. The basic principle of the finite element method is to view each domain as consisting of many small subdomains of various shapes. These subdomains are called finite elements. When meshing in COMSOL Multiphysics, the entire computational domain is decomposed into selectable primitives. The quality of the meshing primitives directly determines the computational accuracy of the finite element simulation. Generally, the finer the meshing, the more accurate the calculation result. However, too fine a grid makes the calculation amount very complex. Since the simplified geometric model was relatively regular, free triangular meshes were used to construct the vibrating membrane. The whole structure was divided by the sweep function, and the divided meshes did not appear as irregularly shaped meshes, which made the calculation results more accurate.

**Figure 3:**
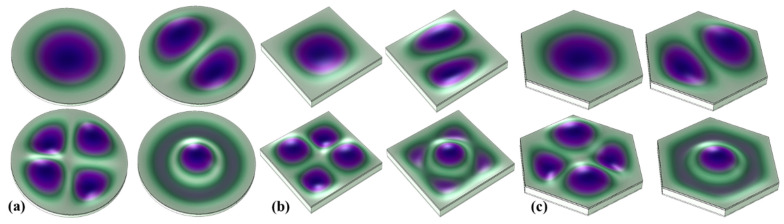
The first four vibration modes of PMUT. (**a**) Circular diaphragm; (**b**) square diaphragm; (**c**) hexagonal diaphragm.

The resonant frequency of the transducer and the vibration modes at different resonant frequencies were analyzed. Figure 3 shows the first four vibration modes of the three diaphragm transducers. It can be seen that the three structural mode shapes generally had the same trend. In the first-order mode, the whole film vibrated up and down, the center amplitude was the largest, and the edge amplitude was the smallest. In the second-order mode, the film was divided into two parts, in the radial direction and in the opposite, vibration direction. In the third-order mode, the film was equally divided into four parts along the radial direction; the vibration trends of the diagonal part was the same, and the vibration trends of the adjacent regions are opposite. In the fourth-order mode, the center and edge of the film vibrated in opposite directions.

The resonance frequency values of the first four orders of the circular diaphragm transducer were obtained through simulation, as shown in Table 3. Compared with the theoretically calculated value, it can be seen that the simulation result was very close to the theoretical value. The error was relatively small, and the simulation results had reference values. As the resonance order increased, the error gradually decreased. This may have been due to the simplification of the computational model during the finite element simulation.

### 3.3. Static Analysis

**Figure 4:**
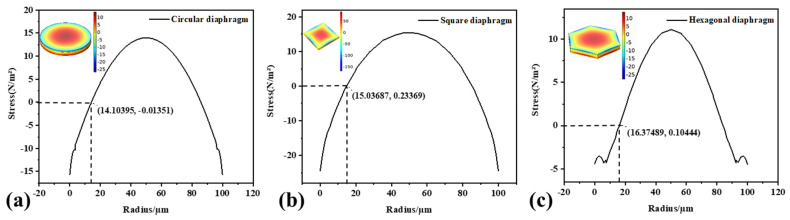
Membrane stress distribution under uniform pressure of 1 Pa. (**a**) Round film; (**b**) square film; (**c**) hexagonal film.

When bending vibration occurs in the fundamental mode, the strain varies in different regions. As the membrane bends upwards, the upper surface expands near the center and contracts near the edges. Finite element simulation software is used to simulate the stress variation of circular, square, and hexagonal surfaces. A uniform pressure load was applied to the membrane to deform it in the fundamental-mode shape. From the equivalent stress nephogram of the film, it can be seen that the stress polarity was positive and negative, reflecting the film from compression to tension, and vice versa. The three structures’ stress distribution curves are shown in Figure 4. The polarities at the center and edge of the film were opposite, and the maximum stress occurred at the center and edge of the film. The maximum displacement occurred at the center of the film, and the displacement curve along the centerline of the film was parabolic. The trends in the three structures were consistent. For this purpose, the upper electrode should cover an area with the same polarity in the center. The top electrode edge should be covered where the stress value is zero to avoid charge neutralization and maximize the output voltage. From the stress distribution curve, the area where the stress was zero was approximately 70% of the film area.

**Figure 5:**
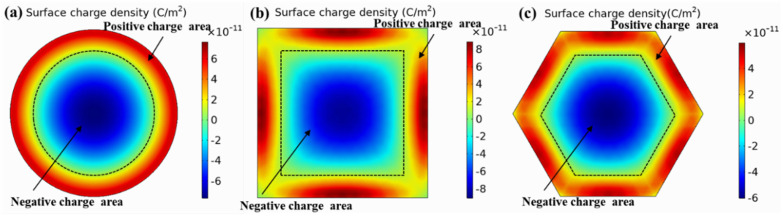
Membrane surface charge density distribution. (**a**) Round membrane; (**b**) square membrane; (**c**) hexagonal membrane.

Figure 5 shows the surface charge density distribution of the film. When a uniform load is applied to the piezoelectric film, due to the piezoelectric effect, the polarities of the charges induced on the surface of the film are not the same. To obtain the maximum amount of charge, the upper electrode should be set to the same shape as the membrane. The edge of the upper electrode should be covered where the charge is zero to maximize the acquisition of the charge signal. The dotted line in the figure indicates the edge where the surface charge was zero. The area where the charge was zero was also approximately 70% of the film area, which was consistent with the stress distribution on the film surface and achieved mutual verification. Therefore, the radius or side length r of the upper electrode should satisfy r=0.7a.

## 4. Structure Optimization

To improve the performance of the transducer for high-frequency applications, the critical structural dimensions of the sensor were optimized, the core structural parameters were extracted, and the corresponding multi-parameter-combination efficient-optimization design method was developed.

### 4.1. Optimization of Film Radius or Side Length

**Figure 6:**
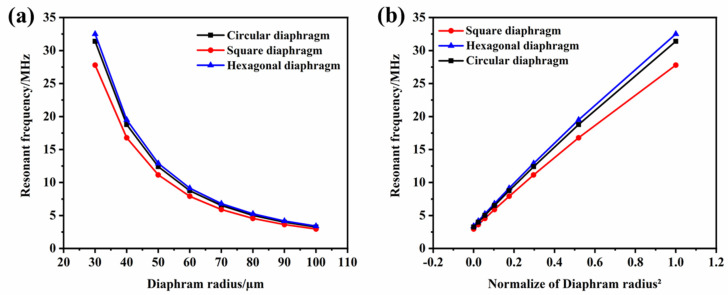
(**a**) The relationship between the device radius and the resonant frequency; (**b**) the relationship between the reciprocal of the square of the normalized device radius and the resonant frequency.

The resonant frequency of the transducer is closely related to the device material and structural parameters. The structural parameters of the device are mainly the thickness and side length or the radius of the vibrating membrane. The material properties were determined by the MEMS standard micromachining process, so the preferred resonant frequency could be obtained by varying the radius and thickness for different applications. The resonant frequencies of the three structures at different radii were simulated and compared. As shown in Figure 6, within the same device radius, the resonant frequency of the hexagonal film was the highest, followed by the traditional circular film, and the square film was the lowest. At high frequencies, the magnitude of the frequency boost was more pronounced. Furthermore, as the radius increased, the resonant frequency of the transducer decreased gradually. According to Figure 6b, the resonant frequency of the device was inversely proportional to the square of the film radius, which is consistent with Equation (5).

### 4.2. Optimization of Film Thickness

**Figure 7:**
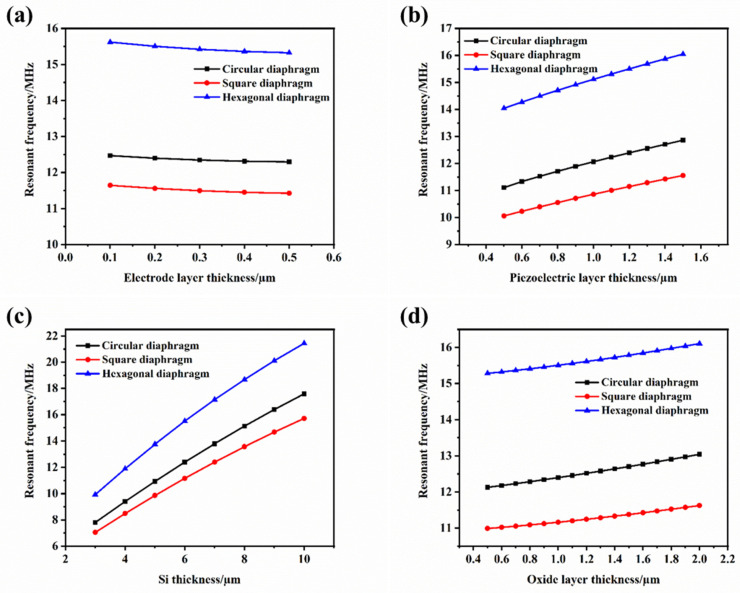
The effect of structural layer thickness on the resonant frequency. (**a**) Relationship between electrode layer thickness and resonant frequency; (**b**) relationship between piezoelectric layer thickness and resonant frequency; (**c**) relationship between silicon layer thickness and resonant frequency; (**d**) relationship between buried oxygen layer thickness and resonant frequency.

It can be seen from Equation (5) that the resonant frequency of the device is proportional to its thickness during bending vibration. The thickness of the film was simulated and analyzed by the finite element simulation method. The thicknesses of the diaphragm piezoelectric layer, the electrode layer, the structural layer Si, and the buried oxide layer, respectively, were. For devices with the same radius, increases in the film thickness of the three structures can improve the resonant frequency of the device, as shown in Figure 7. Furthermore, it can be seen from the figure that when the thickness of the structure was the same, the characteristic frequency of the hexagonal structure was higher than that of the circle and the square.

**Figure 8:**
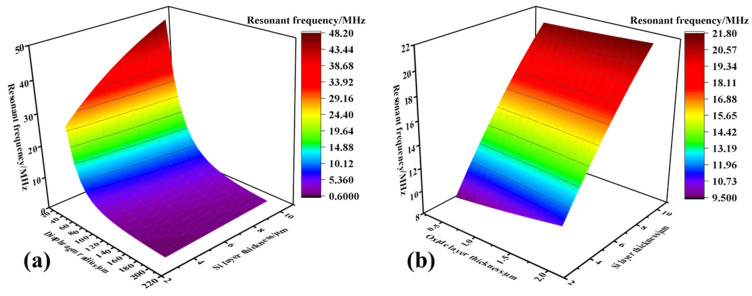
A three-dimensional diagram of the effect of the structure layer’s size on the resonant frequency of the device. (**a**) Resonant frequency versus diaphragm radius and Si layer thickness; (**b**) resonant frequency versus oxide layer thickness and Si layer thickness.

To analyze the mechanism through which the influence of the multi-parameter interaction on the resonance frequency was exerted, a three-dimensional drawing was created. As shown in Figure 8a, the lateral dimension was much larger than the thickness dimension, so the influence of the membrane radius on the resonant frequency was relatively higher than that of the membrane thickness. Meanwhile, as shown in Figure 8b, since the thickness of the silicon of the device layer was much greater than that of the structural oxide layer, the silicon layer had a great influence on the resonant frequency of the device. Since the thickness of the oxide layer has little effect on improving the structural performance, it should be as small as possible according to the actual process limit. The resonant frequency range of the structure is determined by setting the thickness of the silicon layer and the lateral dimension of the film. To achieve a frequency higher than 10 MHz, considering the influence of the process, when the thickness of the silicon of the device layer is set to 5 μm, the radius of the film should satisfy a ≤50 μm. In addition, the dimensions of both should be adjusted for optimal performance within practical process constraints.

**Figure 9:**
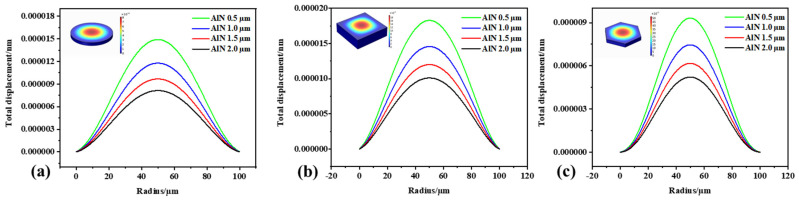
The surface displacement distribution of the membrane when it vibrates. (**a**) Circular diaphragm; (**b**) square diaphragm; (**c**) hexagonal diaphragm.

The film’s response was analyzed, and Figure 9 shows the film displacement distribution curve. The displacement was highest at the center point of the film. As the film thickness increased, the displacement of the film gradually decreased. At the same size, the displacement at the center point of the square film was higher than that of the circle and the hexagon. This may have been because the square vibrating diaphragm had the largest effective area, while the hexagonal vibrating diaphragm had the smallest effective area.

## 5. Results and Discussion

### 5.1. Sensitivity and Linearity

**Figure 10:**
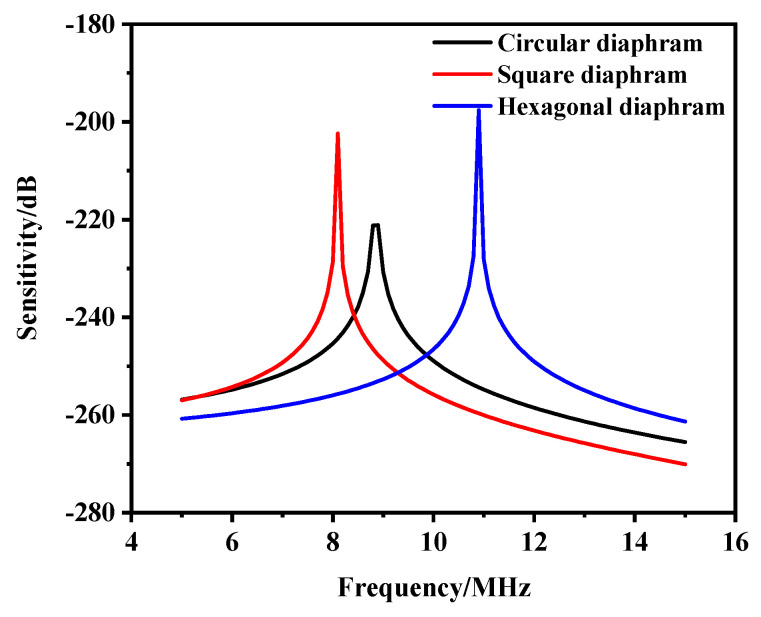
Three structural sensitivity simulation curves.

The frequency sweep range was set to 5–15 MHz in the COMSOL finite element analysis software, and the sweep interval was 10 kHz. At the same size and with a uniform load of 1 Pa, the sensitivity curves of the three mode-shape transducers calculated by Equation (9) are shown in Figure 10. It can be seen from the figure that the resonant frequency of the hexagonal diaphragm was the highest, and the sensitivity of the structure was also the highest at the resonant fundamental frequency. The square vibration resonance frequency was the lowest, but the sensitivity of the square film was better than that of the round film. Therefore, designing the receiving transducer with a hexagonal diaphragm offers a significant advantage in obtaining high sensitivity at the microscale.

**Figure 11:**
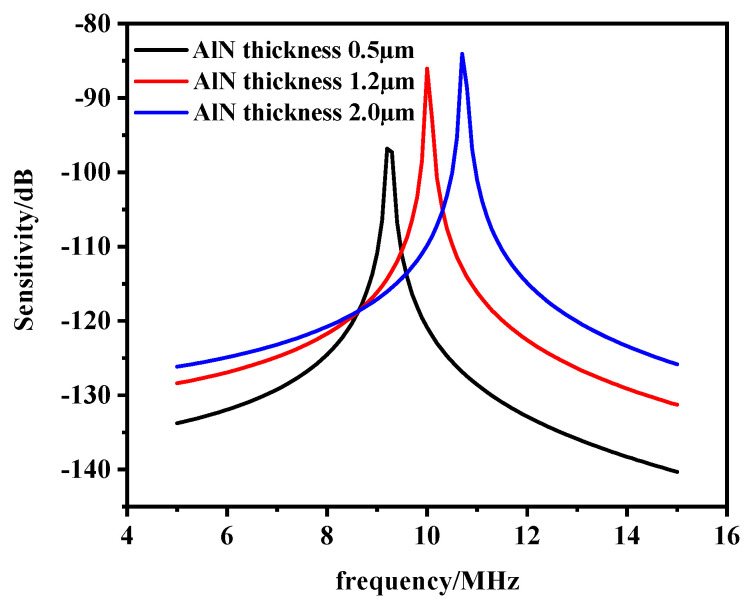
Sensitivity curves of different piezoelectric layer thicknesses.

The effect of the thickness of the piezoelectric layer on the sensitivity was analyzed by parametric sweep, as shown in Figure 11. It can be concluded that the sensitivity at the resonant frequency increased with the increase in the thickness of the piezoelectric layer. Excessive film thickness may result in a stress mismatch. Therefore, considering the preparation process, a film thickness of 1.2 μm can be selected.

**Figure 12:**
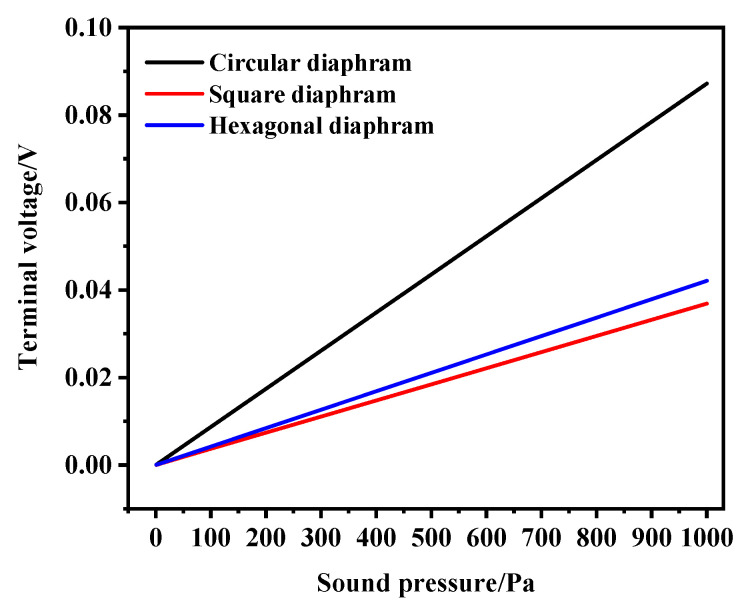
Three structural linearity simulation curves.

The linearity of the PMUT was simulated and analyzed. The sound pressure scanning range was set at 0–1000 Pa and the scanning interval was set at 100 Pa. As can be seen from Figure 12, the linearity of the transducer was good.

### 5.2. Analysis of External Sound Pressure

**Figure 13:**
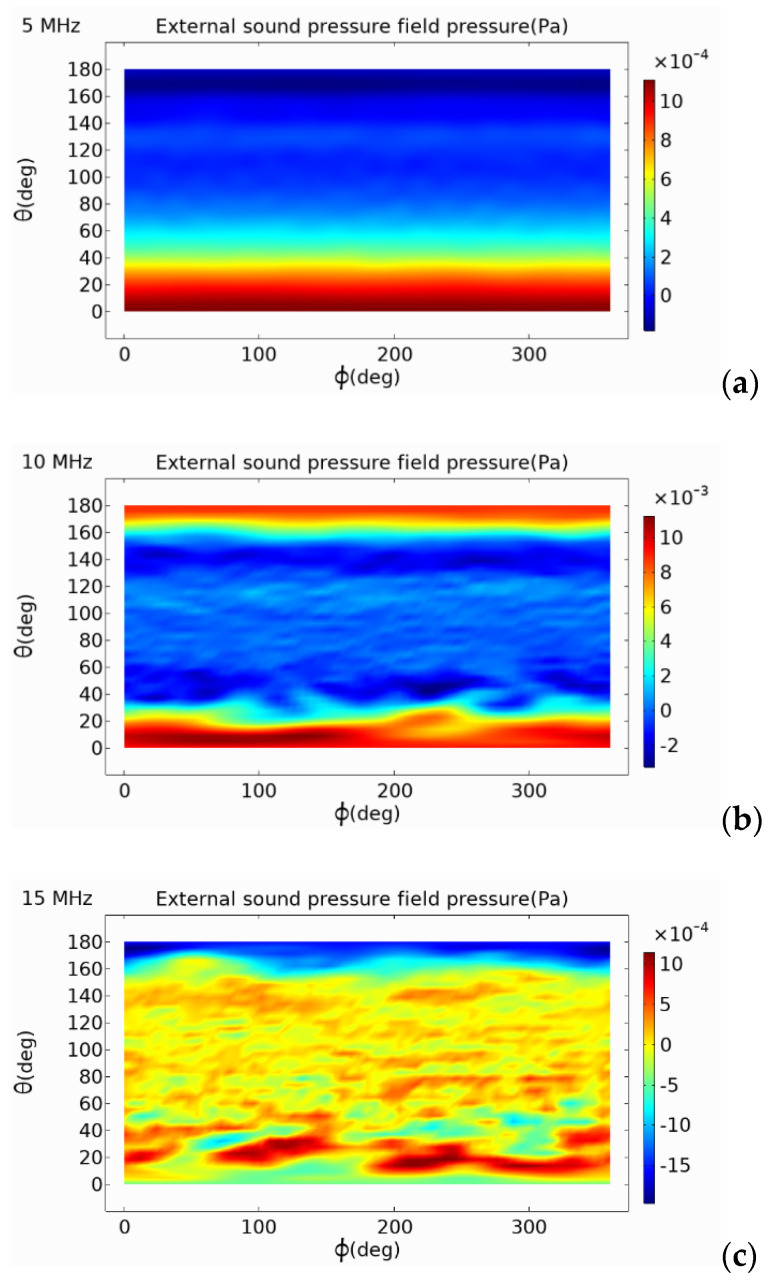
Far-field sound pressure of circular-diaphragm PMUT at different frequencies. (**a**) 5 MHz; (**b**) 10 MHz; (**c**) 15 MHz.

**Figure 14:**
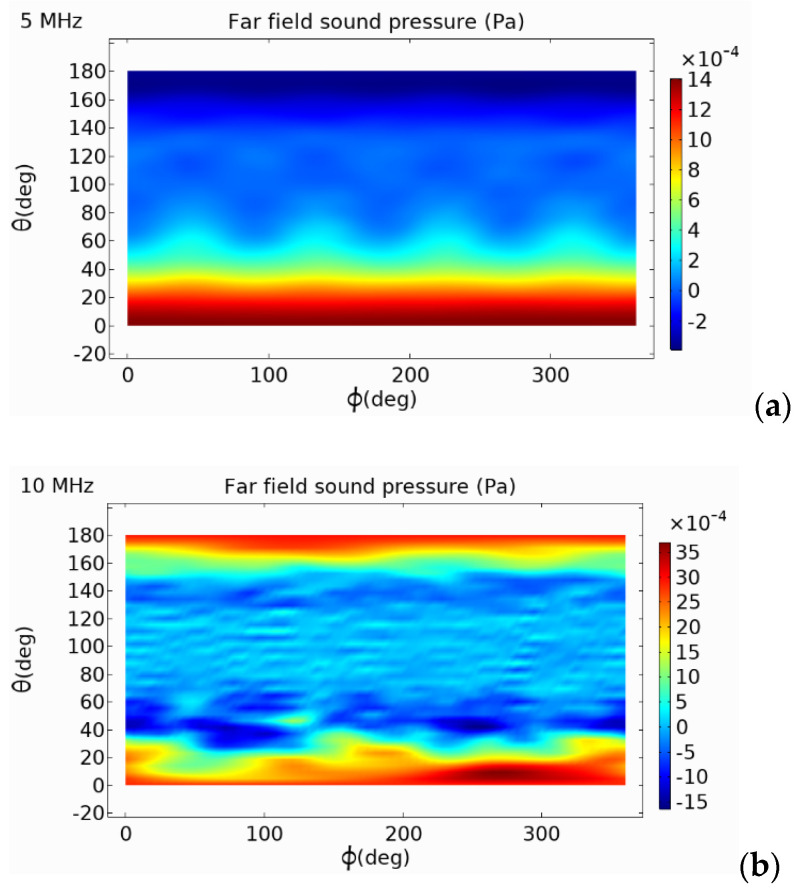

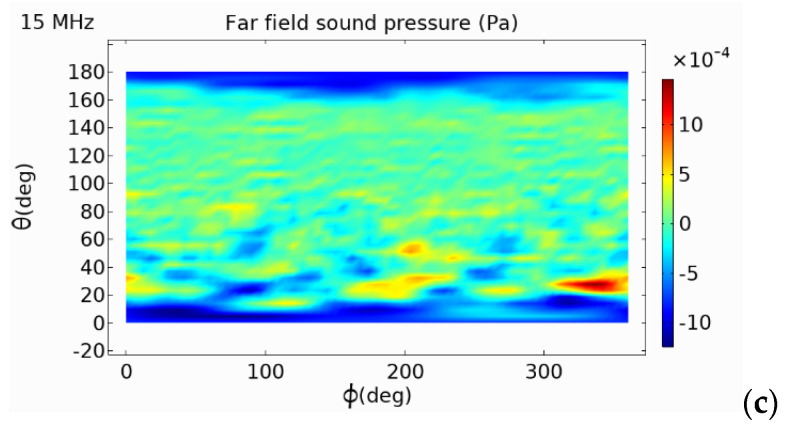
Far-field sound pressure of square-diaphragm PMUT at different frequencies. (**a**) 5 MHz; (**b**) 10 MHz; (**c**) 15 MHz.

**Figure 15:**
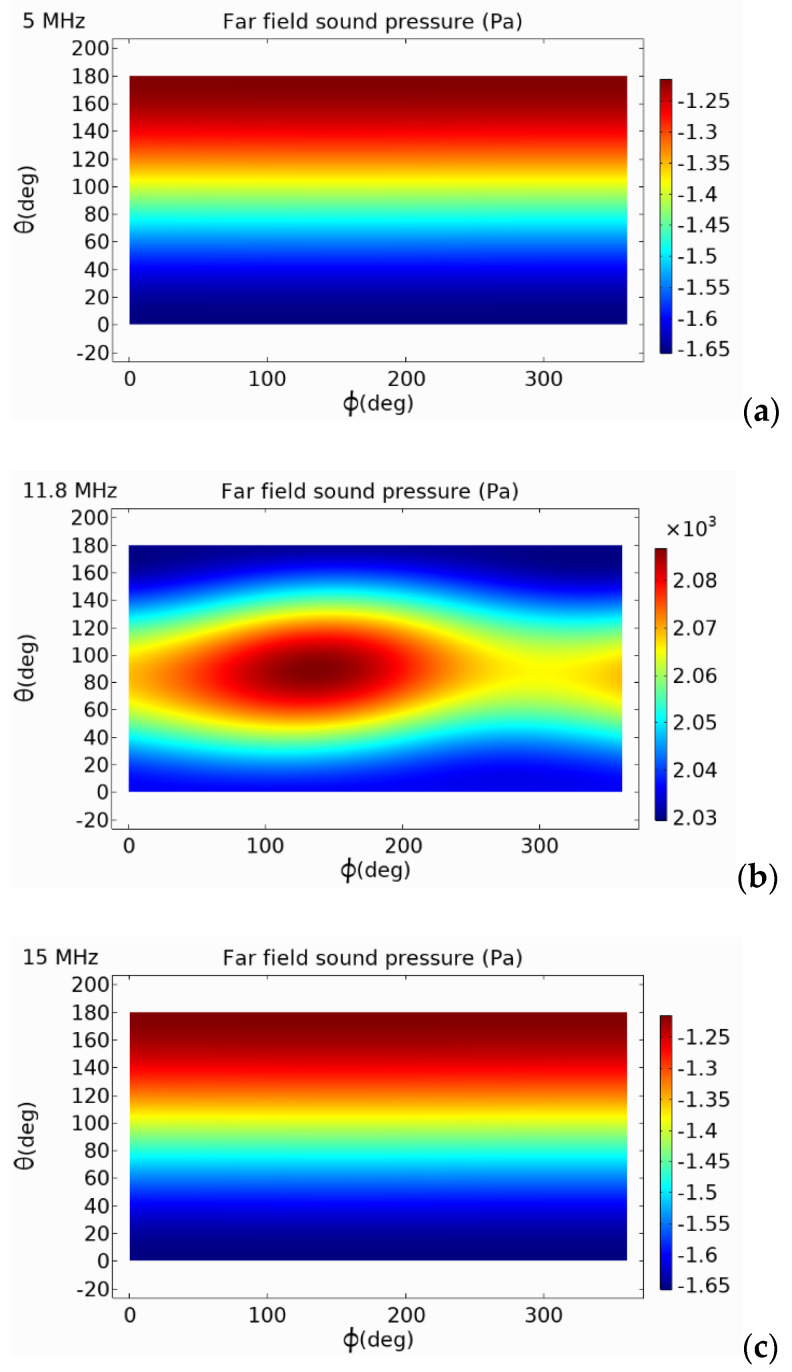
Far-field sound pressure of hexagonal-diaphragm PMUT at different frequencies. (**a**) 5 MHz; (**b**) 11.8 MHz; (**c**) 15 MHz.

In the finite element simulation model, an air domain was established. Through the coupling of pressure acoustics and solid mechanics, the emission performance of the circular, square, and hexagonal PMUTs were analyzed. From the two-dimensional external field sound pressure diagram shown in Figure 13, Figure 14 and Figure 15, it can be seen that the external field sound-pressure level of the PMUT was highest at the resonant frequency. At low frequencies, the sound field transmission was regular. At high frequencies, the transmissions of the PMUT in the acoustic domain may interfere with each other. At the same size, since the resonant frequency of the hexagon is higher than that of the circle, in a certain receiving area, the hexagonal PMUT has a strong penetration ability, and the penetration depth is better than that of the other shapes. It can be seen from the two-dimensional external field pressure diagram of the structure that compared with the circular and square membranes, the radiated sound pressure crosstalk of the hexagonal membrane was smaller. Furthermore, at the resonant frequency, the radiated sound pressure field was concentrated at a point, resulting in the maximum external field pressure value of the hexagonal membrane being much higher than that of the circular membrane.

**Figure 16:**
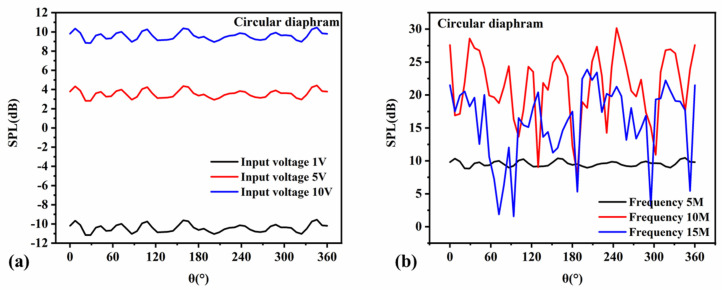
Circular-diaphragm far-field sound-pressure level curves. (**a**) Far-field sound-pressure level curves at different input voltages; (**b**) far-field sound-pressure level curves at different frequencies.

The far-field sound-pressure level value of the circular transducer was analyzed. It can be seen from Figure 16a that with the increase in the voltage, the far-field sound-pressure level output of the transducer also increased gradually, showing a positive correlation. From Figure 16b, it can be concluded that the far-field SPL output of the transducer was highest at the resonant frequency. At low frequencies, the directionality of the sound-wave transmission was good, and the far-field sound-pressure level in all directions was relatively uniform. At high frequencies, the sound-pressure level produced crosstalk, and the curve fluctuated more obviously.

**Figure 17:**
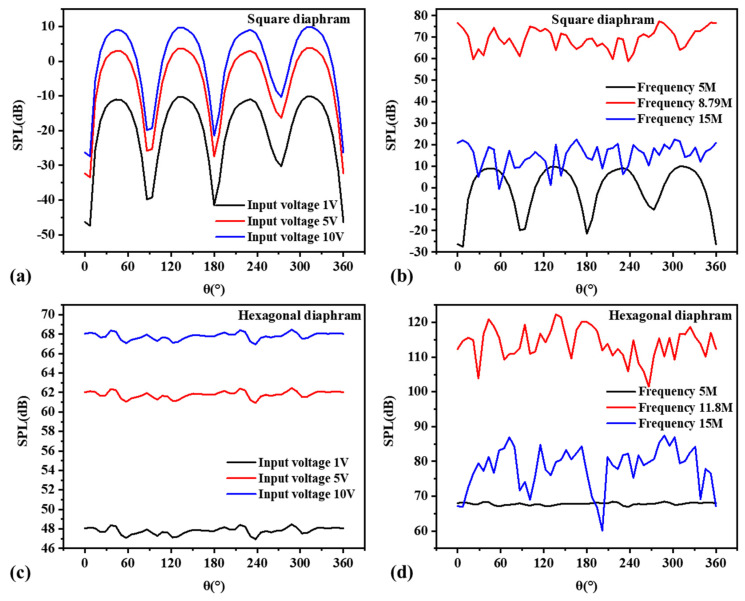
Far-field sound-pressure level curves of square and hexagonal diaphragms. (**a**) The relationship curve between the input voltage of the square diaphragm and the sound-pressure level; (**b**) the relationship between the frequency of the square diaphragm and the sound-pressure level; (**c**) the relationship between the input voltage and the sound-pressure level of the hexagonal diaphragm; (**d**) the relationship between the frequency of the hexagonal diaphragm and the sound-pressure level.

The same analysis method was used to analyze the far-field sound pressure values of the square-diaphragm and hexagonal-diaphragm PMUT, as shown in Figure 17. The far-field sound-pressure level of the hexagonal diaphragm was the highest. The sound-pressure level curve of the square diaphragm was affected by the shape of the film and fluctuated greatly, and the directivity of the film was poorer than that of the circle diaphragm.

**Figure 18:**
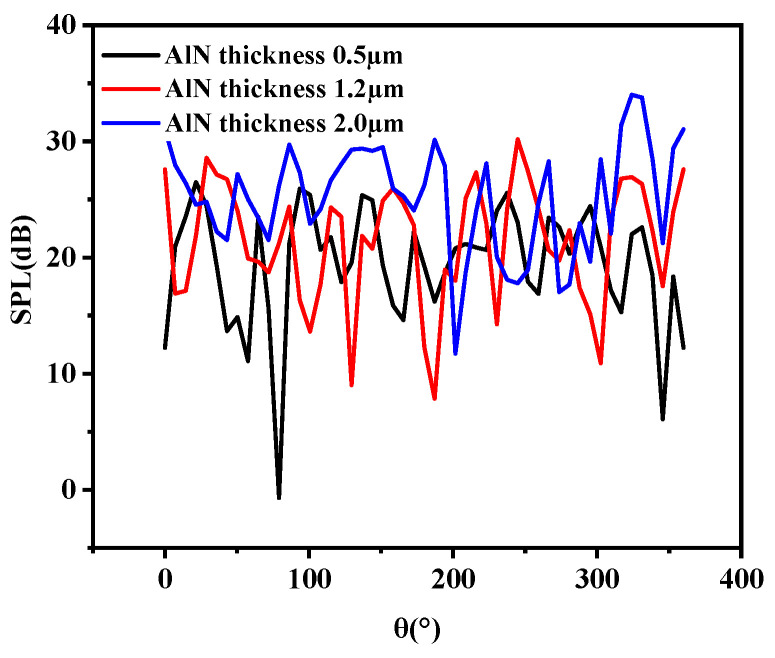
Far-field sound-pressure levels of circular diaphragm PMUTs at different piezoelectric layer thicknesses.

The emission sound-pressure level curves at the resonance frequency of the circular thin film under different piezoelectric layer thicknesses were analyzed by finite element parametric scanning, as shown in Figure 18. It can be seen that the higher the thickness, the higher the emission sound-pressure level, and that the higher the thickness of AlN, the smaller the fluctuation of the sound-pressure level curve.

**Figure 19:**
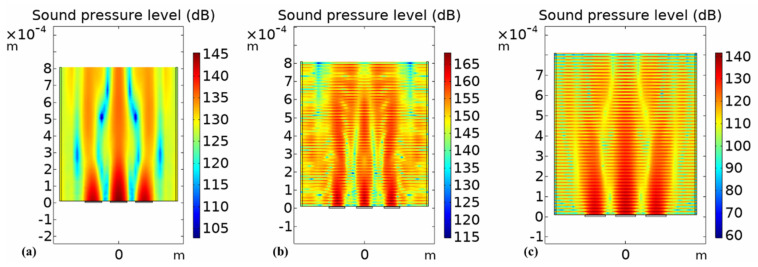
External-field sound-pressure level radiation pattern of the array. (**a**) Circular diaphragm; (**b**) hexagonal diaphragm; (**c**) square diaphragm.

A 3 × 3 array finite element model was established, and the array performances of the three modal PMUTs were compared and analyzed, as shown in Figure 19. A voltage of 10 V was input to the upper and lower electrodes of each array element to analyze the sound pressure transmission performance of the array at the first-order resonant frequency. As can be seen from the figure, the output sound pressure of the array along the axis of the vertical structure was higher. Furthermore, compared with the single array element, the sound-pressure level value of the external field was significantly improved in the array form. Therefore, a multi-array element design can be used to effectively improve PMUT performance. In addition, the sensor can be phased in the later stage to reduce the coupling between the array elements and produce the beam scanning, deflection, and focusing of the ultrasonic wave.

The comparison of the indicators of the three types of PMUT is shown in Table 4. Among them, the external field pressure displayed the maximum value at the resonance frequency. A high resonant frequency in a small size can be produced by using a hexagonal structure. In addition, the sensitivity and emission sound-pressure level of the PMUT was improved due to the change in the diaphragm. At the same size, compared to the circular diaphragm, the sensitivity of the square diaphragm increased by 8.5%, and the sensitivity of the hexagonal diaphragm increased by 10.7%. The maximum emission sound-pressure level of the hexagonal diaphragm was 6.6 times higher than that of the circular diaphragm.

### 5.3. Fabrication and Discussions

**Figure 20:**
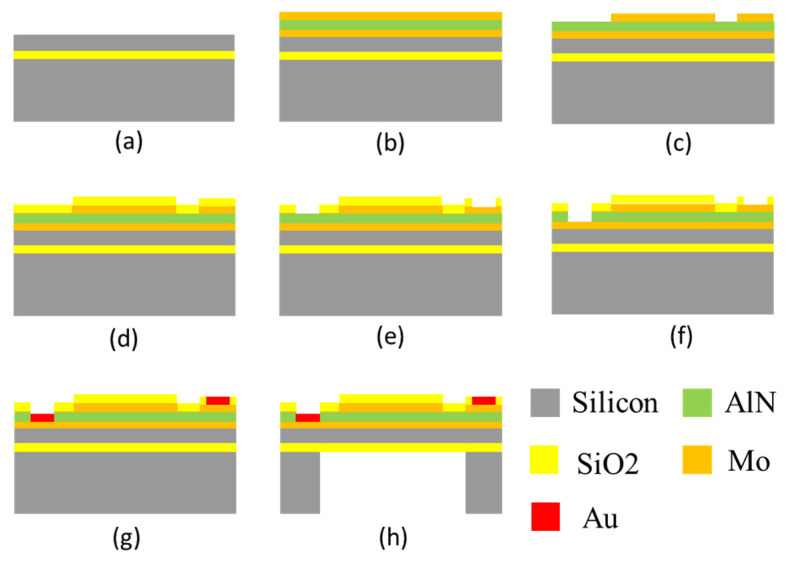
(**a**–**h**) Schematic diagram of PMUT preparation process.

According to the structural design scheme described in Section 2.1, the process preparation process was designed, as shown in Figure 20. First, a Mo/AlN/Mo electrode layer and a piezoelectric layer of 0.2 μm/1 μm/0.2 μm were sequentially grown on the SOI silicon wafer by magnetron sputtering, as shown in Figure 20b. The Mo upper electrode was then patterned using ion-beam etching (IBE), as shown in Figure 20c. A protective oxide layer was deposited by PECVD and etched, as shown in Figure 20d,e. Next, the aluminum nitride piezoelectric layer was wet-etched, as shown in Figure 20f. Subsequently, Au was deposited at the upper and lower electrodes using a lift-off method for subsequent wiring, as shown in Figure 20g. Finally, DRIE was used to etch the back cavity to release the vibrating membrane.

The use of three-mask PMUT micromachining technology with deep silicon etching as the core improves the process consistency and yield and is expected to produce low-cost, mass-manufactured sensors. In addition, current sensor research mostly focuses on the low-frequency band. By optimizing its design, the transmission performance of the sensor can be improved to help with high-frequency ultrasound applications.

Compared with previous studies, this paper has the following advantages: (1) Finite element modeling was performed using boundary conditions. The finite element simulation model was simplified, and the amount of calculation was reduced; (2) SOI substrate was used as the substrate, and the preparation process was relatively simple. The fabrication cost can be reduced, and the fabrication consistency was high; (3) multimodal design optimization provides ideas for improving sensor performance; (4) our study provides a method for analyzing the transmission performance of high-frequency PMUTs.

## 6. Conclusions

In this paper, the design of an AlN-based high-frequency air-backed ultrasonic transducer was described. A three-dimensional finite element model of the air-coupled backplane PMUT was established. Three typical diaphragm shapes were designed: circular, square, and hexagonal. Using the multi-physics finite element simulation method, the optimal performance of the PMUT vibrating membrane design at a specific frequency was studied. The parametric scanning of the three key dimensions of the structure showed that the fundamental resonance frequency, emission sound-pressure level, and sensitivity of the hexagonal diaphragm were higher than those of the circular diaphragm at the same size. PMUTs designed with new piezoelectric materials and piezoelectric structures are helpful in research on high-frequency ultrasound applications. So far, we have designed the sensor fabrication process based on the finite element optimization results and started fabrication experiments. In the future, we will prepare sensors and test their performance for comparative analysis. Furthermore the impedance matching of the sensor when the application medium is different will be considered in terms of both the structural design and the packaging design. In addition, the use of Sc-doped AlN piezoelectric materials will be considered to continue to optimize the performance of the sensor.

## Figures and Tables

**Table 1:** The finite element model parameters of the PMUT.

Parameters	Description
Diaphragm shape	Round
Diaphragm radius	50 μm
Piezoelectric layer thickness	1.2 μm
Electrode thickness	0.2 μm
SiO_2_ thickness	1 μm
Si thickness	6 μm

**Table 2:** Material Properties for Finite Element Analysis.

Property	Symbol	AlN	Si	Mo	SiO_2_
Density (kg/m^3^)	ρ	3300	2329	10200	2200
Poisson ratio	v	0.3	0.28	0.31	0.17
Young’s modulus (GPa)	γ	330	170	312	70

**Table 3:** Comparison of theoretical and simulation results of resonant frequencies.

	First Order	Second Order	Third Order	Fourth Order
Theory	11.087 MHz	22.840 MHz	37.471 MHz	42.725 MHz
Simulation	12.290 MHz	24.361 MHz	37.639 MHz	42.815 MHz
Error	1.203 MHz	1.521 MHz	0.168 MHz	0.090 MHz

**Table 4:** Comparison of output performance of PMUTs with the same structure size (a = 50 μm, input voltage 10 V).

Diaphragm	Resonant Frequency (MHz)	Output Voltage (mV)	Center Displacement (nm)	Sensitivity (dB)	Maximum Emission Sound-Pressure Level (dB)	Maximum External Pressure (Pa)
Circular	8.8	0.088	0.0000149	−221.2	10.45	0.01
Square	8.1	0.037	0.0000183	−202.4	9.90	0.0035
Hexagon	10.9	0.042	0.0000093	−197.6	68.47	2080

## Data Availability

The data presented in this study are available on request from the corresponding author.
